# *Helicobacter pylori* Virulence Factors and Clarithromycin Resistance-Associated Mutations in Mexican Patients

**DOI:** 10.3390/pathogens12020234

**Published:** 2023-02-02

**Authors:** Judit Alarcón-Millán, José Bonilla-Delgado, Gloria Fernández-Tilapa, Nayeli Goreti Nieto-Velázquez, Mónica Sierra-Martínez, Víctor Manuel Alvarado-Castro, Enoc Mariano Cortés-Malagón

**Affiliations:** 1 Clinical Research Laboratory/Biomolecules Research Laboratory, Facultad de Ciencias Químico-Biológicas, Universidad Autónoma de Guerrero, Chilpancingo 39070, Mexico; 2 Departament of Biotechnology, Escuela de Ingeniería y Ciencias, Instituto Tecnológico y de Estudios Superiores de Monterrey, Toluca 50110, Mexico; 3 Research Unit, Hospital Regional de Alta Especialidad de Ixtapaluca, Ixtapaluca 56530, Mexico; 4 Research Division, Hospital Juárez de México, Mexico City 07760, Mexico; 5 Centro de Investigación de Enfermedades Tropicales, Universidad Autónoma de Guerrero, Acapulco 39640, Mexico; 6 Genetic Laboratory, Hospital Nacional Homeopático, Mexico City 06800, Mexico

**Keywords:** *Helicobacter*, clarithromycin, resistance, 23S rRNA, *vacA*, *cagA*

## Abstract

Persistent infection with *Helicobacter pylori (H. pylori)* is an important factor in gastric diseases. The vacA and cagA virulence factors of *H. pylori* contribute to the development of these diseases. Triple therapy containing clarithromycin has been used to eradicate this infection. Unfortunately, resistance to this antibiotic is the primary cause of treatment failure. This study aimed to determine the prevalence of clarithromycin resistance-associated mutations and to assess the relationship between virulence factors and Mexican patients infected with *H. pylori*. The *cagA* and *vacA* genotypes were determined by multiplex PCR. Furthermore, a qPCR was used to identify mutations of the 23S rRNA gene. This study reported a prevalence of 84.3% of *H. pylori* among patients with gastric diseases, and the *vacA s1m1/cagA+* genotype was the most frequent (44.8%) in antrum and corpus. Analysis of the 23S rRNA gene revealed a 19.8% prevalence of clarithromycin resistance-associated mutations. The most prevalent mutations were A2143G (56%) and A2142C (25%). A significant association (*p < 0.05*) between the A2142G and the *vacA s1m1/cagA+* genotype was detected. In conclusion, we report a high prevalence (>15%) of clarithromycin resistance-associated mutations, and we found an association between the genotypes of virulence factors and a mutation in the 23S rRNA gene.

## 1. Introduction

*Helicobacter pylori* (*H. pylori*) is a widely distributed bacterium; according to a 2018 meta-analysis, the global prevalence was 44.3% [1,2]. This infection is common in gastrointestinal diseases, from gastritis to gastric cancer [3]. *H. pylori* has a set of factors that facilitate its persistence in the stomach, ensure its survival, and induce interactions with host cells. Virulence factors are associated with an increased risk of peptic ulcer, gastric adenocarcinoma, or MALT-type lymphoma. In addition, other factors (host inflammatory response, host genetic diversity, and environmental factors) are associated with the development of gastric disease [4,5,6]. The vacA virulence factor is a pore forming toxin and its gene has variable structures in the signal region (*s*), *s1* or *s2*; intermediate (*i*), *i1* or *i2*; and the middle region (*m*), *m1* or *m2*. The *s1* and *m1* genotype has been subclassified into three subtypes *s1a*, *s1b*, and *s1c* and *m1a*, *m1b*, and *m1c*, respectively [3]. Recently, two additional regions have been identified, the deletion region (*d*) and the *c* region (*c*). The *d* region is located between the *i* and *m* regions. It is classified as a *d1* or *d2* genotype (with a deletion of 69–81 bp). The *c* region includes the deletion of 15 bp located at the 3′ end of the *vacA* and is divided into *c1* (with deletion) and *c2* (without deletion) [7]. Another important virulence factor is cagA; the strains that express cagA are associated with an increased risk of gastric cancer [3,8]. 

Eradicating this infection markedly reduces the progression or recurrence of these gastric diseases. The first line of treatment for *H. pylori* elimination consists of a triple therapy containing clarithromycin and a proton pump inhibitor (PPI). Unfortunately, the resistance to clarithromycin has increased [9]. Furthermore, clarithromycin resistance varies depending on the geographical region [10]. In Asia, such as South Korea (60%), China (52%), Japan (31%), and southern Europe (25%), the prevalence is higher, while in northern Europe (7%) and Latin America (12%), the prevalence is lower [11,12,13]. 

In regions where clarithromycin resistance is >15%, the treatment guidelines recommend the use of bismuth quadruple therapy (PPI, bismuth, tetracycline, and metronidazole); levofloxacin quadruple therapy (PPI, levofloxacin, amoxicillin, and bismuth); or non-bismuth therapy, which includes sequential, concomitant, and hybrid therapies; among these, concomitant therapy (PPI, amoxicillin, clarithromycin, and metronidazole prescribed at the same time) for 14 days is preferred. In recent years, the use of dual (amoxicillin and vonoprazan) or triple (amoxicillin, clarithromycin, and vonoprazan) therapies has been introduced [12,14].

The mechanism of resistance to clarithromycin observed in *H. pylori* is due to point mutations in the 23S ribosomal RNA sequence in the 50S subunit [15]. The most frequent mutation detected in this sequence is A2143G (80–90%), followed by A2142G (16–17%), and A2142C (2–4%) [10,16], and, less frequently, the mutations A2142C, A2143C, and A2144G [17]. Furthermore, mutations A2115G, G2141A, A2144T, and T2289C have been reported to confer resistance to clarithromycin, while mutations C2694A and T2717C have been associated with low resistance levels [18]. Other mutations, such as G1939A, C2147G, G2172T, T2215C, and C2245T, have been identified; however, their role in clarithromycin resistance is unknown [19].

Studies concerning the association between *H. pylori vacA/cagA* genotypes and clarithromycin resistance are insufficient and remain controversial. Karabiber et al. and Agudo et al. demonstrated that *vacA s1c* and *vacA s2m2* genotypes were more likely to lead to clarithromycin resistance [20,21], and Elviss et al. reported that the *vacA s1m2* genotype is more susceptible but not with either *vacA s1m1* or *vacA s2m2* [22]. It is important to note that in these studies, the association between specific mutations in the 23s rRNA gene and the *vacA/cagA* genotypes of *H. pylori* was not reported.

In Mexico, clarithromycin is still widely used and there are very few reports of resistance to clarithromycin and its associated mutations. This study aimed to determine the prevalence of infection, the 23S rRNA domain V mutations associated with clarithromycin resistance, and the relation between virulence factors in *H. pylori*-infected Mexican patients. Our data showed a high prevalence of *H. pylori* infection among patients with gastric diseases, with a high frequency of mutations associated with the resistance to clarithromycin, and the mutation A2142G was associated with the *vacA s1m1/cagA+* genotype.

## 2. Materials and Methods

### 2.1. Study Population, Gastric Biopsies, and DNA Extraction

The study’s participants were recruited from the endoscopy department at the Hospital Juárez de Mexico in Mexico City. The criteria for selecting patients were as follows: adults (18 to 80 years old) who had not received antibiotic treatment to eradicate *H. pylori*, PPIs, or gastric pH-neutralizing agents during the 15 days before the endoscopic procedure. Excluded patients from the study were patients with endoscopy contraindications, who recently ingested NAIDS and/or antibiotics, and patients with severe concomitant diseases. Written informed consent was obtained from all participants. Finally, 108 patients were included, and four gastric biopsies (two from the antrum, two from de corpus) were obtained from each patient by an endoscopic procedure. Two biopsies were fixed with 4% PFA for histopathology analysis; the other two were transported in 120 µL of cold sterile 1× PBS; and tissue was recovered from PBS with sterile forceps, which was immediately frozen in liquid nitrogen and finally crushed. Biopsies that were not processed were stored at 70 °C until DNA extraction. According to the manufacturer, nucleic acid extraction was performed using the Quick-DNA Microprep Plus Kit (Zymo Research; Irving, CA, USA). The Research and Bioethics Committee of the Hospital Juárez de México approved this work (ethical approval code: HJM 2260/13-A), and each patient signed an informed consent form.

### 2.2. Detection of Helicobacter Pylori and Virulence Markers by PCR

**Figure 1:**
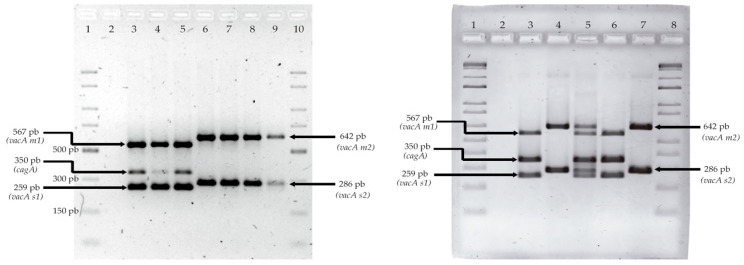
Agarose gel electrophoresis and ethidium bromide were used to visualize multiplex PCR products from the *H. pylori cagA* gene and *vacA* genotypes. Left gel; lane 1 and 10, molecular weight ladder (mwl); lane 2, negative control; lane 3, positive control (43504); lane 4 and 5, *vacA s1m1/cagA+* genotype; and lane 6–9, *vacA s1m1/cagA-* genotype. Right gel; lane 1 and 8, mwl; lane 2, ng; lane 3 and 4 positive controls (43504 and Tx30a); lane 5, *vacA s1m1/s2m2/cagA+* genotype; lane 6, *vacA s1m1/cagA+* genotype; and lane 7, *vacA s2m2/cagA-*.

*Helicobacter pylori* DNA was detected by PCR that was targeting the 16S rRNA gene. The 25 µL PCR mixture contained 200 ng DNA template, 1.5 mM MgCl_2_, 200 µM dNTPs, 0.2 µM each primer, and 1U Taq DNA polymerase. The thermal cycling conditions were 95 °C for 5 min, 30 cycles of 95 °C for 1 min, 58 °C for 1 min, 72 °C for 1 min, and a final extension at 72 °C for 5 min. The *H. pylori* virulence markers were determined by multiplex PCR using primers targeting the *cagA* and *vacA s/m* regions [23]. Briefly, the master mix included 100–200 ng of DNA, 2.5 pmol of primers to target *vacA s1/s2*, 25 pmol of primers to target *vacA m1/m2*, 10 pmol of primers to target *cagA*, 0.25 mM of each dNTPs, 1 U of Taq DNA polymerase, and 1.5 mM of MgCl_2_. All PCR products were visualized by applying agarose gel (1.5%) electrophoresis and staining with ethidium bromide (Figure 1). The thermal cycling profiles were the same as those mentioned above. DNA from the *H. pylori* 43504 (*vacA s1m1/cagA+*) and Tx30a (*vacA s2m2/cagA-)* strains (kindly donated by Gloria Fernández-Tilapa) were used as a positive control in all reactions. All primer sequences are described in Table 1.

### 2.3. Determination of Clarithromycin Resistance Mutations by qPCR

A qPCR was performed to detect 23S rRNA gene point mutations associated with clarithromycin resistance. Primers targeting the 23S rRNA gene and TaqMan™ MGB probes (Applied Biosystems, Waltham, MA, USA) were used (Table 1) to identify the mutations A2142G, A2142C, A2143C, A2143G, and A2144G. Briefly, the 25 µL PCR mixture contained 200 ng DNA template, 1.5 mM MgCl_2_, 200 µM dNTPs, 0.2 µM primers, 0.1 µM wild type VIC-probe, 0.1 µM FAM-modified probe, and 1.5 U Taq DNA polymerase. The assays for each mutation were performed separately. The PCR mixtures were assayed with the following thermal cycling conditions using StepOne™ Real-Time PCR System (Applied Biosystems, Waltham, MA, USA): 95 °C for 5 min, 45 cycles of 95 °C for 30 s, and 58 °C for 40 s. Experiments were carried out in duplicates and DNA from 700392 (ATCC 26695) and 43,504 (ATCC 11637) strains of *H. pylori* were used as the positive controls, sterile deionized water and DNA from W3110 strain of *E. coli* were used as negative controls. Data were analyzed with StepOne version 2.3 software.

### 2.4. Statistical Analysis

Quantitative data from the statistical analysis were presented as mean, standard deviation (SD), range, or percentages. The association between *H. pylori* resistance to clarithromycin and virulence genotypes was analyzed using Fisher’s exact test using ggplot2 software. A *p* < 0.05 was considered statistically significant.

## 3. Results

In this study, 108 patients were enrolled, 67.6% (73/108) were females, and 32.4% (35/108) were males, with an age mean of 52.3 ± 14.4 years (range 18–88 years). With respect to endoscopy and histopathology findings: 10.2% (11/108) of the patients were diagnosed with dyspepsia, 15.7% (17/108) with gastroesophageal reflux disease (GERD), with 27.8% (30/108) as acute gastritis, and 46.3% with (50/108) as chronic gastritis.

The 84.3% (91/108) were positive for *H. pylori*; according to the anatomical site of each patient, 90.1% (82/91) were positive in both the antrum and corpus, 5.5% (5/91) were positives uniquely in the antrum, and 4.4% (4/91) occurred in the corpus. Concerning gastrointestinal disease, 29.7% (27/91) in acute gastritis, 51.6% (47/91) in chronic gastritis, 7.7% (7/91) in dyspepsia, and 11% (10/91) in GERD were positive for *H. pylori*. Table 2 summarizes some characteristics of the *H. pylori-*positives patients in different clinical diagnosis groups.

Concerning the combination of *H. pylori cagA* and *vacA* genotypes by gastric anatomical site, we analyzed the antrum and corpus biopsies from patients infected with *H. pylori*. We observed that of the 91 patients positive for *H. pylori,* 73.6% (67/91) had an identical *vacA/cagA* genotype in both the antrum and corpus. Of these, *vacA s1m1/cagA+* was found in 44.8% (30/67), *vacA s1/cagA+* was detected in 13.4% (9/67), and *vacA s1m1/cagA*− in 10.4% (7/67). The clinical findings showed that 52.2% of these patients were diagnosed with chronic gastritis and 30% with acute gastritis (Table 3).

On the other hand, 16.5% (15/91) had different *vacA/cagA* genotypes in the antrum and corpus. Furthermore, in 5.5% (5/91) of the patients, the *vacA/cagA* genotypes were determined only in the antrum because they were negative for *H. pylori* in the corpus; while in 4.4% (4/91), the *vacA/cagA* genotypes were determined in the corpus because they were negative for *H. pylori* in the antrum (Table 4). In 37.5% (9/24) of these patients, the *vacA s1m1/cagA+* genotype was detected exclusively in the antrum; *vacA s1/cagA+ and vacA s1/cagA−* were most frequent in the corpus, 20.8% (5/24) and 16.6% (4/24), respectively (Table S1). Similarly, these patients were mostly diagnosed with chronic gastric disease.

**Figure 2:**
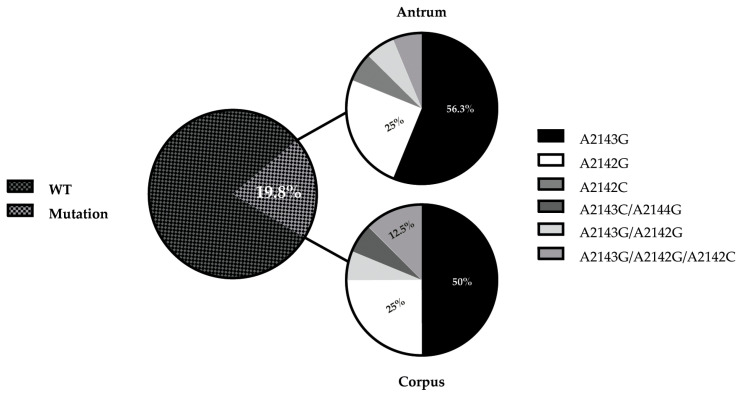
Profile of 23S rRNA mutations in *H. pylori-*positive antrum and corpus biopsies (A2143G, A2142G, A2142C, and A2144G represent clarithromycin resistance-associated mutations).

An analysis of the 23S rRNA gene revealed a prevalence of 19.8% (18/91) of clarithromycin resistance-associated mutations (Clr-ram). According to the biopsy region, for 56.2% (9/16) of patients, *H. pylori* with the A2143G mutation was detected in the antrum and 50% (8/16) was detected in corpus. A2142G represented 25% (4/16) in both the antrum and corpus, and 6.2% (1/16) of the A2142C mutation were represented only in an antrum biopsy. Interestingly, mixed mutations A2143G/A2142G (6.2%) in the both antrum and corpus, A2143C/A2144G (6.2%) in the corpus, and A2143G/A2142G/A2142C (12.5%) in the antrum, were also detected (Figure 2 and Table 5).

The association between the Clr-ram and the *cagA/vacA* genotypes was also analyzed. The A2143G mutation was present in most combined *vacA*/*cagA* genotypes detected in this study. The *vacA s1m1/cagA+* genotype exhibited A2143G, A2142G, and mixed mutations. The A2142G mutation was associated with this genotype in both the antrum and the corpus (*p* = 0.019 and *p* = 0.003, by Fisher’s exact test, respectively). Furthermore, the A2143G mutation was detected in the *vacA s2m2/cagA−* genotype (Table 5).

## 4. Discussion

The present study aimed to determine the frequency and type of mutations in the 23S rRNA gene of *Helicobacter pyl*ori detected in Mexican patients. Furthermore, we investigated the association between *vacA*/*cagA* genotypes and 23S rRNA mutations.

We found a high prevalence of *H. pylori* (84.3%) in patients with gastric disease. This result is in agreement with other studies: in southern Mexico, 76.5% (150/196) of *H. pylori* was reported in gastric biopsies [26]; furthermore, in an age-adjusted analysis, 80% of adults 25 years and older were infected [2]. In general, the prevalence of the *H. pylori* infection is high in Latin American adults: in Mexico, it ranges from 70% to 90%; in Guatemala, 65%; in Chile, 70–90%; and in Brazil, 82% [27].

*H. pylori* has different virulence factors, of which *cagA* and *vacA* are the most studied. Several studies have reported the heterogeneity of the *H. pylori cagA/vacA* genotype [28,29]. In this study, the most frequent genotype was *vacA s1m1/cagA+,* in both the antrum and corpus. Other studies performed in southern Mexico reported 71.1% and 69.7% [30,31]; furthermore, by gastric pathology, the prevalence of this genotype was 70.7% in chronic gastritis, 57.9% in gastric ulcers, and 81.3% in gastric cancer [32]. These findings suggest that the *vacA s1m1/cagA+* genotype predominates in the Mexican population. It is essential to note that the percentages of the *vacA/cagA* genotype combinations can vary by geographical region. For example, in Wenzhou, China, the genotype *vacA s1m1/cagA+* was 90.9% [33]; in southern Vietnam, it was 51.5% [34], and while in northern Spain, it was found in 20.6% and 54.5%, due to mild and severe disease, respectively [35]. Similarly, when the combination of the *s* and *m* alleles of *vacA* is analyzed, the prevalence also differs. The *vacA m1* allele is common in North Asian countries, including Japan and South Korea, whereas the *m2* allele is predominant in Southeast Asia, including Taiwan, China, and Vietnam [36]. Interestingly, in this study, the *vacA s1m1/s2m2/cagA+* genotype in both the antrum and corpus was detected in one patient, and the *vacA s1m1/s1m2/cagA−* genotype was detected in the corpus in two patients. Mixed genotypes have already been reported [37,38], but this event is occasional. On the other hand, we did not detect the *s* or *m* allele in some cases, as reported in other Mexican populations [39].

The detection of antimicrobial resistance to *H. pylori* is usually based on culture approaches (E test or agar dilution method) [40]. However, the special conditions required for specimen transport, growth, and time-consuming processes make them challenging to implement in research laboratories in developing countries. Therefore, it has been necessary to implement molecular tests. PCR-based approaches have been used as alternative assays. They are fast to perform, accurate, and are used directly on different biological samples [41,42]. Some studies have compared the agreement between the culture and the qPCR. For example, Monno et al. reported an 80.6% concordance between the qPCR and the E-test for detecting clarithromycin resistance in gastric biopsies, and Bimaeil et al. reported a significant concordance between these methods (Kappa = 0.85) [43,44]. The Maastricht V/Florence consensus report has already recognized that detecting genes and mutations associated with antibiotic resistance using molecular technologies is a valuable tool for detecting gastric biopsy specimens directly [14]. Therefore, we decided to determine the Clr-ram in the domain V of the 23S rRNA gene in *H. pylori* using a qPCR assay.

Mutations conferring resistance to clarithromycin were detected in 19.8% (18/91) of patients harboring *H. pylori*, and A2143G (56.2%) and A2142G (25%) were the most frequent. These results differ slightly from those reported by Alarcón-Millán et al. They reported 12.5% of the A2143G mutation in the southern Mexico population [30]. Furthermore, in a different region of Mexico, A2143G (57.1%) and A2142G (14.3%) were reported [45]. On the other hand, the prevalence of mutations in other regions of the world is also different. For example, the prevalence of mutations was 37.7% in the Korean population, with A2143G in 90.3% of cases, A2142G in 8.0% and A2142G/A2143G (mixed mutations) represented in 1.7% of cases [46]. In China, the prevalence of A2143G varies between 10–14% [47]; in Malaysia, it was 90.5% [48]; in Iran, A2143G fluctuates between 47.1–68.7% and A2142G 33.3–5.6% [49,50,51]; and in Brazil, the A2143G mutation was the most prevalent (77.8%) [52]. Despite the heterogeneity in the prevalence of 23S rRNA gene mutations of *H. pylori* in different regions, the A2143G mutation remains the most common. In this study, we also detected mixed mutations in three patients; similar results have been reported in other studies [53,54]. Probably in these patients, the presence of heteroresistant strains of *H. pylori* is suggested; therefore, the efficacy of clarithromycin therapy could be diminished.

Some authors have analyzed the virulence genes and clarithromycin resistance, and it has been suggested that *H. pylori vacA* and *cagA* genotypes affect the eradication rates of bacteria [55,56]. Our study reported that the *vacA s1m1/cagA*+ genotype was associated with the A2142G mutation. This result is not in agreement with Agudo et al., who reported that clarithromycin resistance in *H. pylori* isolates was strongly associated with the *vacA s2/m2* genotype; however, no association with 23S rRNA gene mutations and *vacA/cagA* genotypes is reported [21]. Otherwise, different studies have not found an association between *vacA* genotypes and antibiotic resistance [57,58]. Therefore, multicenter studies are needed to study the relationship between clarithromycin resistance-associated mutations and *vacA/cagA* genotypes of *H. pylori.*

There are some limitations to this study. First, no culture-based clarithromycin susceptibility tests were conducted. Second, only *vacA/cagA* genotypes and 23s rRNA classical point mutations were determined, and the mutations were not corroborated by sequencing; third, small sample size.

In conclusion, our data show a high prevalence of *H. pylori* infection among patients with gastric diseases, with a high frequency of mutations associated with resistance to clarithromycin. The A2143G mutation was the most frequent and the A2142G mutation was associated with the *vacA s1m1/cagA+* genotype. These results suggest a change from clarithromycin-based therapy.

## Figures and Tables

**Table 1:** List of primers and sequences of hydrolysis probes used in this study.

Target	Primer/Probe	Size (bp)	Reference
*16S rRNA*	5′-CTGGAGAGACTA AGCCCTCC-3′ 5′-ATTACTGACGCTGATTGTGC-3	109	[24]
*vacA s1/s2*	5′-ATGGAAATACAACAAACACAC-3′ 5′-CTGCTTGAATGCGCCAAAC-3′	259/286	[23]
*vacA m1/m2*	5′-CAATCTGTCCAATCAAGCGAG-3′ 5′-GCGTCAAAATAATTCCAAGG-3′	567/642	[23]
*cagA*	5′-GTTGATAACGCTGTCGCTTC-3′ 5′-GGGTTGTATGATATTTTCCATAA-3′	350	[23]
*23S rRNA*	5′-TCAGTGAAATTGTAGTGGAGGTGAAA-3 5′-CAGTGCTAAGTTGTAGTAAAGGTCCA-3′		[24,25]
Wild type	VIC-AAGACGGAAAGACC-MGBNFQ		
A2142G	FAM-AAGACGGGAAGACC-MGBNFQ		
A2142C	FAM-CAAGACGGCAAGACC-MGBNFQ		
A2143G	FAM-CAAGACGGAGAGACC-MGBNFQ		
A2143C	FAM-CAAGACGGACAGACC-MGBNFQ		
A2144G	FAM-CAAGACGGAAGGACC-MGBNFQ		

**Table 2:** Characteristics of *H. pylori*-infected patients in different clinical diagnosis groups.

Parameter	Clinical Diagnosis *n* = 91			
	AG *n* = 27 (29.7%)	CG *n* = 47 (51.6%)	D *n* = 7 (7.7%)	GERD *n* = 10 (11%)
Mean age ± SD (year)	52.6 ± 13.6	48.9 ± 14.1	57.9 ± 9.8	55.6 ± 14.0
Age range (year)	21–78	18–88	47–71	25–74
Female (%)	19 (20.9)	36 (39.5)	2 (28.6)	7 (7.7)
Male (%)	8 (8.8)	11 (12.1)	5 (71.4)	3 (3.3)

AG = acute gastritis, CG = chronic gastritis, D = dyspepsia, and GERD = gastroesophageal reflux disease.

**Table 3:** Distribution of identical *vacA/cagA* genotypes in both antrum and corpus, and in gastric disease.

Genotype	Antrum/Corpus *n* (%)	Gastric Disease *n*(%)
*vacA s1m1/cagA+*	30 (44.8)	AG 16 (23.8) CG 14 (21.0)
*vacA s1m1/cagA−*	7 (10.4)	AG 1 (1.5) CG 6 (8.9)
*vacA s2m2/cagA+*	1 (1.5)	CG 1 (1.5)
*vacA s2m2/cagA−*	6 (9.0)	CG 4 (6.0) D 1 (1.5) GERD 1 (1.5)
*vacA s1m2/cagA−*	2 (3.0)	CG 2 (3.0)
*vacA s1m1/s2m2/cagA−*	2 (3.0)	CG 2 (3.0)
*vacA s1/cagA+*	9 (13.4)	AG 2 (3.0) CG 4 (6.0) GERD 3 (4.4)
*vacA s1/cagA−*	1 (1.5)	GERD 1 (1.5)
*vacA s1/s2/cagA−*	1 (1.5)	CG 1 (1.5)
*cagA+*	8 (11.9)	AG 1 (1.5) CG 1 (1.5) D 2 (3.0) GERD 4 (5.9)
Total	67 (100)	AG 20 (30.0) CG 35 (52.2) D 3 (4.5) GERD 9 (13.3)

AG = acute gastritis, CG = chronic gastritis, D = dyspepsia, GERD = gastroesophageal reflux disease.

**Table 4:** Distribution of different *vacA/cagA* genotypes by anatomical site and gastric disease of each patient.

	Patient Code	Genotype		Gastric Disease
		Antrum	Corpus	
1	901616	*vacA s1m1/cagA+*	*vacA s1/cagA* *−*	CG
2	901617	*vacA s1m1/cagA+*	*vacA s1/cagA* *−*	CG
3	898796	*cagA* *−*	*vacA s2m2/cagA* *−*	CG
4	898053	*vacA s1/cagA* *−*	*vacA s1m1/s1m2/cagA* *−*	CG
5	905946	*vacA s1m1/s2m2/cagA+*	*vacA s1m1/s2m2/cagA* *−*	CG
6	916426	*vacA s1m1/cagA* *−*	*vacA s1/cagA* *−*	AG
7	920465	*vacA m1/cagA+*	*vacA s1m1/cagA* *−*	D
8	920133	*vacA m1/cagA* *−*	*vacA s1m1/cagA* *−*	AG
9	901618	*vacA s1m1/cagA+*	*vacA s1/cagA* *−*	CG
10	898054	*vacA s1/cagA* *−*	*vacA s1m1/s1m2/cagA* *−*	CG
11	429322	*vacA s1/cagA+*	*vacA s2m2/cagA−*	D
12	924536	*cagA* *−*	*vacA s1/cagA+*	D
13	258914	*vacA s1m1/cagA* *−*	*vacA s1/cagA+*	AG
14	939365	*vacA s1m1/cagA+*	*cagA+*	AG
15	942220	*vacA s1m1/cagA+*	*cagA−*	CG
16	892921	*vacA s1m1/cagA+*	*Hp* (−)	CG
17	936256	*vacA s1m1/cagA+*	*Hp* (−)	AG
18	945229	*vacA s1m1/cagA+*	*Hp* (−)	AG
19	944089	*vacA s1m1/cagA+*	*Hp* (−)	D
20	944392	*cagA+*	*Hp* (−)	GERD
21	57013	*Hp* (−)	*vacA s2m2/cagA* *−*	CG
22	916308	*Hp* (−)	*vacA s1/cagA+*	AG
23	895973	*Hp* (−)	*vacA s1/cagA+*	CG
24	947987	*Hp* (−)	*vacA s1/cagA+*	CG

*Hp* (−) = negative for *H. pylori*, AG = acute gastritis, CG = chronic gastritis, D = dyspepsia, and GERD = gastroesophageal reflux disease.

**Table 5:** *vacA* and *cagA* genotypes and clarithromycin resistance-associated mutations.

Patient Code		23S rRNA Mutations/Genotype	
		Antrum	Corpus
1	886725	A2143G/*vacA s1m1/cagA+*	A2143G/*vacA s1m1/cagA+*
2	913514	A2143G/*vacA s2m2/cagA+*	A2143G*/vacA s2m2/cagA+*
3	787261	A2143G/*vacA s2m2/cagA−*	A2143G/*vacA s2m2/cagA−*
4	919583	A2143G/*vacA s2m2/cagA−*	A2143G/*vacA s2m2/cagA−*
5	804158	A2143G/*vacA s1m2/cagA−*	A2143G/*vacA s1m2/cagA−*
6	945776	A2143G/*vacA s1/cagA+*	A2143G/*vacA s1/cagA+*
7	905946	A2143G/*vacA s1m1/s2m2/cagA+*	A2143G/*vacA s1m1/s2m2/cagA+*
8	939365	A2143G*/vacA s1m1/cagA+*	A2143G/*vacA−/cagA+*
9	897467	A2143G*/vacA s1m2/cagA−*	A2143C/A2144G/*vacA s1m2/cagA−*
10	910108	A2142G/*vacA s1m1/cagA+*	A2142G/*vacA s1m1/cagA+*
11	910242	A2142G*/vacA s1m1/cagA+*	A2142G/*vacA s1m1/cagA+*
12	940196	A2142G/*vacA s1m1/cagA+*	A2142G/*vacA s1m1/cagA+*
13	943311	A2142G/*vacA s1m1/cagA+*	A2142G/*vacA s1m1/cagA+*
14	898796	A2142C*/cagA−*	Wt/*vacA s2m2/cagA−*
15	60702	A2143G/A2142G/*vacA s2m2/cagA−*	A2143G/A2142G/*vacA s2m2/cagA−*
16	0214F	Wt*/cagA+*	A2143G/A2142G/A2142C/*cagA+*
17	936256	A2143G/A2142G/A2142C/*vacA s1m1/cagA+*	*Hp* (−)
18	895973	*Hp* (−)	A2143G/A2142G/A2142C/*vacA s1/cagA+*

A2142G/*vacA s1m1/cagA+* (*p* = 0.019 (antrum), *p* = 0.003 (corpus)). *Hp* (−) = negative for *H. pylori*.

## Data Availability

The data presented in this study are available on request from the corresponding author. The data are not publicly available due to privacy or ethical restrictions.

## Supplementary Materials

The following supporting information can be downloaded at: https://www.mdpi.com/article/10.3390/pathogens12020234/s1, Table S1: Distribution of different vacA and cagA genotypes according to anatomical site and gastric disease.

## References

[1] Zamani M., Ebrahimtabar F., Zamani V., Miller W.H., Alizadeh-Navaei R., Shokri-Shirvani J., Derakhshan M.H. (2018). Systematic review with meta-analysis: The worldwide prevalence of *Helicobacter pylori* infection. Aliment. Pharmacol. Ther..

[2] Hooi J.K.Y., Lai W.Y., Ng W.K., Suen M.M.Y., Underwood F.E., Tanyingoh D., Malfertheiner P., Graham D.Y., Wong V.W.S., Wu J.C.Y. (2017). Global Prevalence of *Helicobacter pylori* Infection: Systematic Review and Meta-Analysis. Gastroenterology.

[3] Salama N.R., Hartung M., Muller A. (2013). Life in the human stomach: Persistence strategies of the bacterial pathogen *Helicobacter pylori*. Nat. Rev. Microbiol..

[4] Wen S., Moss S. (2009). *Helicobacter pylori* virulence factors in gastric carcinogenesis. Cancer Lett..

[5] Nahid-Samiei M., Rahimian G., Shafigh M., Taheri F., Karami-Hurestani M., Sanaei M.J., Heshmati M., Bagheri N. (2020). Enhanced Frequency of CD19(+)IL-10(+)B Cells in Human Gastric Mucosa Infected by *Helicobacter pylori*. Am. J. Med. Sci..

[6] Sanaii A., Shirzad H., Haghighian M., Rahimian G., Soltani A., Shafigh M., Tahmasbi K., Bagheri N. (2019). Role of Th22 cells in *Helicobacter pylori*—Related gastritis and peptic ulcer diseases. Mol. Biol. Rep..

[7] Trang T.T.H., Binh T.T., Yamaoka Y. (2016). Relationship between vacA Types and Development of Gastroduodenal Diseases. Toxins.

[8] Ohnishi N., Yuasa H., Tanaka S., Sawa H., Miura M., Matsui A., Higashi H., Musashi M., Iwabuchi K., Suzuki M. (2008). Transgenic expression of *Helicobacter pylori* CagA induces gastrointestinal and hematopoietic neoplasms in mouse. Proc. Natl. Acad. Sci. USA.

[9] Leja M., Grinberga-Derica I., Bilgilier C., Steininger C. (2019). Review: Epidemiology of *Helicobacter pylori* infection. Helicobacter.

[10] Thung I., Aramin H., Vavinskaya V., Gupta S., Park J.Y., Crowe S.E., Valasek M.A. (2016). Review article: The global emergence of *Helicobacter pylori* antibiotic resistance. Aliment. Pharmacol. Ther..

[11] Camargo M.C., Garcia A., Riquelme A., Otero W., Camargo C.A., Hernandez-Garcia T., Candia R., Bruce M.G., Rabkin C.S. (2014). The problem of *Helicobacter pylori* resistance to antibiotics: A systematic review in Latin America. Am. J. Gastroenterol..

[12] Bujanda L., Nyssen O.P., Vaira D., Saracino I.M., Fiorini G., Lerang F., Georgopoulos S., Tepes B., Heluwaert F., Gasbarrini A. (2021). Antibiotic Resistance Prevalence and Trends in Patients Infected with *Helicobacter pylori* in the Period 2013-2020: Results of the European Registry on *H. pylori* Management (Hp-EuReg). Antibiotics.

[13] Megraud F., Bruyndonckx R., Coenen S., Wittkop L., Huang T.D., Hoebeke M., Benejat L., Lehours P., Goossens H., Glupczynski Y. (2021). *Helicobacter pylori* resistance to antibiotics in Europe in 2018 and its relationship to antibiotic consumption in the community. Gut.

[14] Malfertheiner P., Megraud F., Rokkas T., Gisbert J.P., Liou J.M., Schulz C., Gasbarrini A., Hunt R.H., Leja M., O’Morain C. (2022). Management of *Helicobacter pylori* infection: The Maastricht VI/Florence consensus report. Gut.

[15] Francesco V.D., Zullo A., Hassan C., Giorgio F., Rosania R., Ierardi E. (2011). Mechanisms of *Helicobacter pylori* antibiotic resistance: An updated appraisal. World J. Gastrointest. Pathophysiol..

[16] Ansari S., Yamaoka Y. (2022). *Helicobacter pylori* Infection, Its Laboratory Diagnosis, and Antimicrobial Resistance: A Perspective of Clinical Relevance. Clin. Microbiol. Rev..

[17] Harrison U., Fowora M.A., Seriki A.T., Loell E., Mueller S., Ugo-Ijeh M., Onyekwere C.A., Lesi O.A., Otegbayo J.A., Akere A. (2017). *Helicobacter pylori* strains from a Nigerian cohort show divergent antibiotic resistance rates and a uniform pathogenicity profile. PLoS ONE.

[18] Rimbara E., Noguchi N., Kawai T., Sasatsu M. (2008). Novel mutation in 23S rRNA that confers low-level resistance to clarithromycin in *Helicobacter pylori*. Antimicrob. Agents Chemother..

[19] Marques A.T., Vitor J.M.B., Santos A., Oleastro M., Vale F.F. (2020). Trends in *Helicobacter pylori* resistance to clarithromycin: From phenotypic to genomic approaches. Microb. Genom..

[20] Karabiber H., Selimoglu M.A., Otlu B., Yildirim O., Ozer A. (2014). Virulence factors and antibiotic resistance in children with *Helicobacter pylori* gastritis. J. Pediatr. Gastroenterol. Nutr..

[21] Agudo S., Perez-Perez G., Alarcon T., Lopez-Brea M. (2010). High prevalence of clarithromycin-resistant *Helicobacter pylori* strains and risk factors associated with resistance in Madrid, Spain. J. Clin. Microbiol..

[22] Elviss N.C., Owen R.J., Xerry J., Walker A.M., Davies K. (2004). *Helicobacter pylori* antibiotic resistance patterns and genotypes in adult dyspeptic patients from a regional population in North Wales. J. Antimicrob. Chemother..

[23] Chattopadhyay S., Patra R., Ramamurthy T., Chowdhury A., Santra A., Dhali G.K., Bhattacharya S.K., Berg D.E., Nair G.B., Mukhopadhyay A.K. (2004). Multiplex PCR assay for rapid detection and genotyping of *Helicobacter pylori* directly from biopsy specimens. J. Clin. Microbiol..

[24] Kargar M., Doosti A., Ghorbani-Dalini S. (2013). Detection of four clarithromycin resistance point mutations in *Helicobacter pylori*: Comparison of real-time PCR and PCR-RFLP methods. Comp. Clin. Pathol..

[25] De Francesco V., Zullo A., Ierardi E., Giorgio F., Perna F., Hassan C., Morini S., Panella C., Vaira D. (2010). Phenotypic and genotypic *Helicobacter pylori* clarithromycin resistance and therapeutic outcome: Benefits and limits. J. Antimicrob. Chemother..

[26] Roman-Roman A., Giono-Cerezo S., Camorlinga-Ponce M., Martinez-Carrillo D.N., Loaiza-Loeza S., Fernandez-Tilapa G. (2013). vacA genotypes of *Helicobacter pylori* in the oral cavity and stomach of patients with chronic gastritis and gastric ulcer. Enferm. Infecc. Microbiol. Clin..

[27] World Gastroenterology Organization (2011). World Gastroenterology Organization Global Guideline: *Helicobacter pylori* in developing countries. J. Clin. Gastroenterol..

[28] Idowu A., Mzukwa A., Harrison U., Palamides P., Haas R., Mbao M., Mamdoo R., Bolon J., Jolaiya T., Smith S. (2019). Detection of *Helicobacter pylori* and its virulence genes (cagA, dupA, and vacA) among patients with gastroduodenal diseases in Chris Hani Baragwanath Academic Hospital, South Africa. BMC Gastroenterol..

[29] Oktem-Okullu S., Cekic-Kipritci Z., Kilic E., Seymen N., Mansur-Ozen N., Sezerman U., Gurol Y. (2020). Analysis of Correlation between the Seven Important *Helicobacter pylori* (*H. pylori*) Virulence Factors and Drug Resistance in Patients with Gastritis. Gastroenterol. Res. Pract..

[30] Alarcon-Millan J., Fernandez-Tilapa G., Cortes-Malagon E.M., Castanon-Sanchez C.A., De Sampedro-Reyes J., Cruz-Del Carmen I., Betancourt-Linares R., Roman-Roman A. (2016). Clarithromycin resistance and prevalence of *Helicobacter pylori* virulent genotypes in patients from Southern Mexico with chronic gastritis. Infect. Genet. Evol..

[31] Martinez-Carrillo D.N., Atrisco-Morales J., Hernandez-Pando R., Reyes-Navarrete S., Betancourt-Linares R., Cruz-del Carmen I., Illades Aguiar B., Roman-Roman A., Fernandez-Tilapa G. (2014). *Helicobacter pylori* vacA and cagA genotype diversity and interferon gamma expression in patients with chronic gastritis and patients with gastric cancer. Rev. Gastroenterol. Mex..

[32] Roman-Roman A., Martinez-Carrillo D.N., Atrisco-Morales J., Azucar-Heziquio J.C., Cuevas-Caballero A.S., Castanon-Sanchez C.A., Reyes-Rios R., Betancourt-Linares R., Reyes-Navarrete S., Cruz-Del Carmen I. (2017). *Helicobacter pylori* vacA s1m1 genotype but not cagA or babA2 increase the risk of ulcer and gastric cancer in patients from Southern Mexico. Gut Pathog..

[33] Li Y., Lin R., Jin Y., Jin S., Chen B., Wu X. (2021). Genotyping *Helicobacter pylori* antibiotic resistance and virulence-associated genes in patients with gastric cancer in Wenzhou, China. Arab. J. Gastroenterol..

[34] Nguyen T.H., Ho T.T.M., Nguyen-Hoang T.P., Qumar S., Pham T.T.D., Bui Q.N., Bulach D., Nguyen T.V., Rahman M. (2021). The endemic *Helicobacter pylori* population in Southern Vietnam has both South East Asian and European origins. Gut Pathog..

[35] Fernandez-Reyes M.M., Tamayo E., Rojas-Rengifo D., Fischer W., Carrasco-Garcia E., Alonso M., Lizasoain J., Bujanda L., Cosme A., Montes M. (2019). *Helicobacter pylori* pathogenicity and primary antimicrobial resistance in Northern Spain. Eur. J. Clin. Invest..

[36] Yin L., Liu F., Guo C., Wang Q., Pan K., Xu L., Xiong Y., Chen Y., Chen Z. (2018). Analysis of virulence diversity of 73 *Helicobacter pylori* strains isolated in Guizhou province, China. Mol. Med. Rep..

[37] Queiroz D.M., Silva C.I., Goncalves M.H., Braga-Neto M.B., Fialho A.B., Fialho A.M., Rocha G.A., Rocha A.M., Batista S.A., Guerrant R.L. (2012). Higher frequency of cagA EPIYA-C phosphorylation sites in *H. pylori* strains from first-degree relatives of gastric cancer patients. BMC Gastroenterol..

[38] Secka O., Antonio M., Berg D.E., Tapgun M., Bottomley C., Thomas V., Walton R., Corrah T., Thomas J.E., Adegbola R.A. (2011). Mixed infection with cagA positive and cagA negative strains of *Helicobacter pylori* lowers disease burden in The Gambia. PLoS ONE.

[39] Lopez-Vidal Y., Ponce-de-Leon S., Castillo-Rojas G., Barreto-Zuniga R., Torre-Delgadillo A. (2008). High diversity of vacA and cagA *Helicobacter pylori* genotypes in patients with and without gastric cancer. PLoS ONE.

[40] Arslan N., Yilmaz O., Demiray-Gurbuz E. (2017). Importance of antimicrobial susceptibility testing for the management of eradication in *Helicobacter pylori* infection. World J. Gastroenterol..

[41] Wang Y.H., Wang Y.H., Li Z., Wang L., Zhu-Ge L.Y., Zhao R.L., Wu S., Wang Y., An Y., Xie Y. (2018). A systematic review and meta-analysis of genotypic methods for detecting antibiotic resistance in *Helicobacter pylori*. Helicobacter.

[42] Tshibangu-Kabamba E., Yamaoka Y. (2021). *Helicobacter pylori* infection and antibiotic resistance—from biology to clinical implications. Nat. Rev. Gastroenterol. Hepatol..

[43] Monno R., Giorgio F., Carmine P., Soleo L., Cinquepalmi V., Ierardi E. (2012). *Helicobacter pylori* clarithromycin resistance detected by Etest and TaqMan real-time polymerase chain reaction: A comparative study. APMIS.

[44] Binmaeil H., Hanafiah A., Mohamed Rose I., Raja Ali R.A. (2021). Development and Validation of Multiplex Quantitative PCR Assay for Detection of *Helicobacter pylori* and Mutations Conferring Resistance to Clarithromycin and Levofloxacin in Gastric Biopsy. Infect. Drug Resist..

[45] Camorlinga-Ponce M., Gomez-Delgado A., Aguilar-Zamora E., Torres R.C., Giono-Cerezo S., Escobar-Ogaz A., Torres J. (2020). Phenotypic and Genotypic Antibiotic Resistance Patterns in *Helicobacter pylori* Strains from Ethnically Diverse Population in Mexico. Front. Cell Infect. Microbiol..

[46] Kim S.Y., Park J.M., Lim C.H., Lee H.A., Shin G.Y., Choe Y., Cho Y.K., Choi M.G. (2021). Types of 23S Ribosomal RNA Point Mutations and Therapeutic Outcomes for *Helicobacter pylori*. Gut Liver.

[47] Liu Z., Shen J., Zhang L., Shen L., Li Q., Zhang B., Zhou J., Gu L., Feng G., Ma J. (2008). Prevalence of A2143G mutation of *H. pylori*-23S rRNA in Chinese subjects with and without clarithromycin use history. BMC Microbiol..

[48] Alfizah H., Norazah A., Hamizah R., Ramelah M. (2014). Resistotype of *Helicobacter pylori* isolates: The impact on eradication outcome. J. Med. Microbiol..

[49] Keshavarz Azizi Raftar S., Moniri R., Saffari M., Razavi Zadeh M., Arj A., Mousavi S.G., Mirzaei Ghazi Kalayeh H., Dastehgoli K. (2015). The *Helicobacter pylori* resistance rate to clarithromycin in Iran. Microb. Drug Resist..

[50] Yousefi A., Eslami S., Noorbakhsh S., Haghighi M., TaheriNia L., Ehsanipour F., Ashouri S. (2019). The Resistance Rate of *Helicobacter pylori* to Clarithromycin and Main Mutations on Bacterial Genomic Responsible for Bacterial Resistance: A Comparative Study in Children and Adults, Tehran and Iran. Infect. Disord. Drug Targets.

[51] Vazirzadeh J., Falahi J., Moghim S., Narimani T., Rafiei R., Karbasizadeh V. (2020). Molecular Assessment of Resistance to Clarithromycin in *Helicobacter pylori* Strains Isolated from Patients with Dyspepsia by Fluorescent In Situ Hybridization in the Center of Iran. Biomed. Res. Int..

[52] Martins G.M., Sanches B.S., Moretzsohn L.D., Lima K.S., Cota B.D., Coelho L.G. (2016). Molecular Detection of Clarithromycin and Fluoroquinolones Resistance in *Helicobacter pylori* Infection, Directly Applied to Gastric Biopsies, in an Urban Brazilian Population. Arq. Gastroenterol..

[53] Krashias G., Bashiardes S., Potamitou A., Potamitis G.S., Christodoulou C. (2013). Prevalence of *Helicobacter pylori* cagA and vacA genes in Cypriot patients. J. Infect. Dev. Ctries..

[54] Seo S.I., Do B.J., Kang J.G., Kim H.S., Jang M.K., Kim H.Y., Shin W.G. (2019). *Helicobacter pylori* Eradication According to Sequencing-Based 23S Ribosomal RNA Point Mutation Associated with Clarithromycin Resistance. J. Clin. Med..

[55] Mi M., Wu F., Zhu J., Liu F., Cui G., Wen X., Hu Y., Deng Z., Wu X., Zhang Z. (2021). Heterogeneity of *Helicobacter pylori* Strains Isolated from Patients with Gastric Disorders in Guiyang, China. Infect. Drug Resist..

[56] Sugimoto M., Yamaoka Y. (2009). Virulence factor genotypes of *Helicobacter pylori* affect cure rates of eradication therapy. Arch. Immunol. Ther. Exp..

[57] Dai J., Zhao J., Mao L., Hu Y., Lv B. (2022). Study on the value of antibiotic-resistant gene detection in *Helicobacter pylori* in China. Exp. Ther. Med..

[58] Alavifard H., Mirzaei N., Yadegar A., Baghaei K., Smith S.M., Sadeghi A., Zali M.R. (2021). Investigation of Clarithromycin Resistance-Associated Mutations and Virulence Genotypes of *Helicobacter pylori* Isolated from Iranian Population: A Cross-Sectional Study. Curr. Microbiol..

