# Supplementary material for: Helicobacter pylori Virulence Factors and Clarithromycin Resistance-Associated Mutations in Mexican Patients
Source: Pathogens. 2023 Feb 2;12(2):234. doi: 10.3390/pathogens12020234 (PMC9959081; doi:10.3390/pathogens12020234)
Supplement: Supplementary file 1 [file pathogens-12-00234-s001.zip › pathogens-2157537-supplementary.pdf]

Table S1. Distribution of different *vacA* and *cagA* genotypes according to anatomical site and gastric disease.

**Genotype**	**Anatomic site n (%)**		**Gastric disease n(%)**	
	**Antrum**	**Corpus**		
vacA s1m1/cagA+	9 (37.5)		5 (20.8)	CG
			4 (16.7)	AG
vacA s1m1/cagA-	2 (8.3)	2 (8.3)	3 (12.5)	AG
			1 (4.2)	D
vacA s2m2/cagA-		3 (12.5)	2 (8.3)	CG
			1 (4.2)	D
vacA s1m1/s2m2/cagA+	1 (4.2)		1 (4.2)	CG
vacA s1m1/s2m2/cagA-		1 (4.2)	1 (4.2)	CG
vacA s1m1/s1m2/cagA-		2 (8.3)	2 (8.3)	CG
vacA s1/cagA+	1 (4.2)	5 (20.8)	2 (8.3)	CG
			2 (8.3)	AG
			2 (8.3)	D
vacA s1/cagA-	2 (8.3)	4 (16.7)	5 (20.8)	CG
			1 (4.2)	AG
vacA m1/cagA+	1 (4.2)		1 (4.2)	D
vacA m1/cagA-	1 (4.2)		1 (4.2)	AG
cagA+	1 (4.2)	1 (4.2)	1 (4.2)	AG
			1 (4.2)	GERD
cagA-	2 (8.3)	1 (4.2)	2 (8.3)	CG
			1 (4.2)	D
Hp (-)	4 (16.6)	5 (20.8)	4 (16.7)	CG
			3 (12.5)	AG
			1 (4.2)	D
			1 (4.2)	GERD
